# Citizen Science and the Urban Ecology of Birds and Butterflies — A Systematic Review

**DOI:** 10.1371/journal.pone.0156425

**Published:** 2016-06-10

**Authors:** James Wang Wei, Benjamin P. Y-H. Lee, Low Bing Wen

**Affiliations:** 1 National Parks Board, Singapore, Singapore; 2 Department of Geography, National University of Singapore, Singapore, Singapore; 3 Durrell Institute of Conservation & Ecology, School of Anthropology & Conservation, University of Kent, Canterbury, United Kingdom; University of Bologna, ITALY

## Abstract

Citizen science has gained widespread currency as a tool for ecological research over the past decade. However, in the discipline of urban ecology, the existing contributions and future potential of citizen science engagement, specifically in terms of knowledge gain, have not yet been comprehensively explored. Here, we present a systematic review of published work on the urban ecology of birds and butterflies in relation to their use of citizen science data between 2005 and 2014. We compared the number of studies that used citizen science data to the number of studies that could potentially have employed data derived from citizen science. The take-up rates of citizen science data were 21% and 26% for birds and butterflies respectively. Most studies that employed citizen science used volunteer-derived data as primary data, and adopted Collegial, Collaborative and Contributional engagement modes to the exclusion of Contractual and Co-created arrangements. There was no evidence that citizen science studies investigated a different organismal scale (community vs. species) compared to the urban ecology literature. For both taxa, citizen science contributions were lower than expected compared to their representation in the urban ecology literature for studies on species-environment relationships at landscape and micro-environment scales, as well as behavioural ecology in general. Other research topics that could benefit from further citizen science involvement include breeding studies and guild analyses for birds, and multi-taxa studies for butterflies. Promising models of citizen science engagement for urban ecology are highlighted in relation to their thematic foci and methodological detail, and a number of research questions that could be productively addressed using citizen science are identified. The dynamics of contemporary engagement between citizen science and urban ecology described by this review could inform the design and refinement of urban ecology–citizen science programmes in order to optimise their scientific contributions.

## 1. Introduction

Citizen science (hereafter known as CS)—the mass involvement of non-professionals in scientific research—has gained significant traction over the past decade as a tool for advancing ecological science [1,2]. The public appeal of observing and monitoring nature at large has spurred the proliferation of CS programmes spanning multiple domains and environmental scales, ranging from broad-scale phenological and biogeographical studies, to monitoring activities which focus on more localised scientific questions such as fauna-environment relationships, plant-animal interactions and behavioural ecology [3]. Although the merits of these datasets for ecological research in general have been well-articulated [4,5], the potential applications of CS projects to contribute specifically to the nascent discipline of urban ecology has not yet been systematically explored.

Urban ecology (hereafter known as UE) in the traditional sense is defined as ecological research conducted in an urban setting [6,7], and aims to describe and explain the processes determining the abundance and distribution of organisms as well as their interactions with one another and their environment [8]. The biological parameters of this definition distinguish it in scope from more recent and rapidly expanding approaches seeking to integrate human and biological aspects of urban ecosystems [9–11]. Three characteristics of UE *sensu stricto* as a field of scientific enquiry make it exceptionally accessible to citizen science involvement: (a) a strong history of amateur interest in biology as a whole [12], (b) the importance of skilled observations for data collection as opposed to specialised equipment [1], and (c) the geographic accessibility of study sites for city dwellers.

This paper examines that first and most fundamental aspect of research for both CS and UE: the choice of a scientific question. It is imperative that CS programmes and projects should define problem statements that are clear, feasible and useful to the scientific community. This inherits broader concerns expressed by scientists over the practical outcomes of general conservation monitoring programmes in relation to their design and focus [13,14]. However, despite the central importance of defining good scientific questions to the enterprise of CS, information relevant to the choice of appropriate research questions in the context of the specific taxa of interest and past relevant research in the field of UE is not readily accessible to designers and implementers of CS programmes. Moreover, given that the definition of research questions in CS is often constrained by engagement modes in terms of programme organization, we sought to characterise potential associations between research themes and engagement modes to facilitate practical application of the themes identified as being potentially important.

Whereas other authors have written about various aspects of the practice and application of CS projects focused on ecology, these have generally addressed other aspects of the CS life-cycle. For example, several excellent reviews of CS in relation to general ecological research exist [15–17]. Much existing work has addressed issues of the design and development of these projects from social [18–20], institutional [21,22] and infrastructural [23] perspectives. Various works have discussed and proposed procedural [24] and statistical [25] approaches for data quality control. Others have examined issues of recruitment and training [26,27]. Another research focus has been evaluating the outcomes of CS programmes, by providing conceptual frameworks [28,29], reviewing the applications of specific types of programmes [30,31], assessing participant outcomes [2] or quantifying scientific outputs [4,5,12]. This work extends the objectives of Lepczyk et al. [32] and Dickinson et al. [15] in seeking to clarify the role of CS in relation to ecology with reference to a specific subset of ecological studies, that of UE. We employ a similar approach adopted by Tulloch et al. [5] in comparing the objectives of CS programmes, but compare two taxa, address a wider range of CS projects not limited to Atlases or Breeding Bird Surveys.

The specific aims of this review were:

1. To identify the main themes open to CS involvement that urban ecological research has addressed in the past decade for birds and butterflies, and to quantify the extent to which CS datasets have actually contributed to these themes;
2. To characterize different paradigms of CS *sensu* Shirk et al. [33] that have emerged in relation to research into the various UE themes;
3. To assess what is known about trends of research efforts by theme, and identify potential themes for CS to generate new knowledge;
4. To discuss the implications of the findings from (I), (II) and (III) on the design of CS programmes for biodiversity monitoring in urban areas.

In 2015, the National Parks Board of Singapore (NParks) launched a range of Community in Nature (CIN) CS programmes to involve the community in biodiversity monitoring and research programmes including the Garden Bird Watch, Butterfly Watch and BioBlitz, as part of the NParks CIN Biodiversity Watch and NParks CIN Biodiversity Survey @ Parks series. Our proximate motivations for this study were thus to understand specific angles of data analysis which would optimise the scientific contributions of these programmes, identify practical options for refining survey protocols in service of specific research objectives, and to target research topics which could be suitable for the development of new programmes. We hope that this review will serve as a useful reference for designers and managers of similar UE-CS programmes of the types of hypotheses that have been valued by the scientific community over the past decade. This knowledge could be further applied to refine survey protocols to optimise both pure and applied scientific outcomes of such programmes.

Birds (Aves) and butterflies (Papilionoidea) were selected as focal taxa for this review for three main reasons: (a) sufficient ecological work has been undertaken for these taxa that broad literature trends may be gleaned over the past decade alone, (b) birds and butterflies, especially the more eurytopic species, are less dependent on the preservation of contiguous tracts of pristine habitat for their persistence, and thus show practical promise for responding positively to targeted landscape interventions in urban areas, (c) they generally hold a cross-cultural appeal for laymen, to the extent that CS programmes involving these taxa may be typically described as enjoying vibrant social support. For example, birds and butterflies were recently shown in a comprehensive database of biodiversity-related citizen science projects to account for the most number of CS projects, relative to other faunal taxa [4].

## 2. Materials and Methods

### a. Search criteria

**Fig 1:**
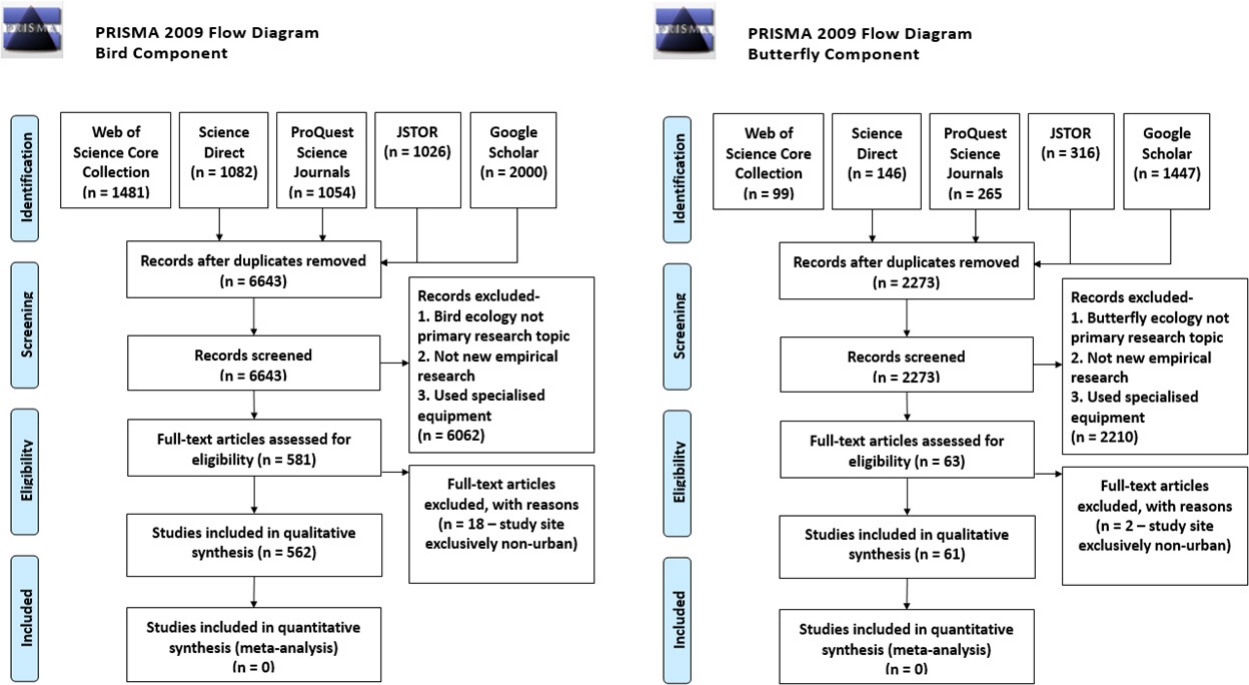
PRSIMA flowchart summarising the workflow adopted in this systematic review.

We searched five databases for journal articles relevant to the practice of UE and CS published from January 2005 to July 2014. General search statistics are reported in accordance to PRISMA (Preferred Reporting Items for Systematic Reviews and Meta-Analysis, www.prisma-statement.org) guidelines in Fig 1 (PRISMA checklist included as S1 Table), while the total numbers of articles reviewed with respect to the four specific search terms are summarised in Table 1. A table detailing the conceptual structure of this research is provided in supplementary materials (S2 Table).

**Table 1:** Search terms, databases and respective article yield applied for this literature review. Numbers in brackets indicate the total number of articles returned, compared to the number available for review (maximum 1000, except for Web of Science) in every case.

Search term/ Database	Web of Science Core Collection	Science Direct	Proquest Science Journals	JSTOR	Google Scholar
"Urban" AND "Bird" AND "Ecology"	1460	1000 (7656)	1000 (3394)	1000 (2025)	1000 (29800)
"Citizen Science" AND "Urban" AND "Bird"	21	82	54	26	1000 (1450)
"Urban" AND "Butterfly" AND "Ecology"	91	114	259	313	1000 (14200)
"Citizen Science" AND "Urban" AND "Butterfly"	8	32	6	3	447

Articles were reviewed for relevance if they contained all of the search terms anywhere in the text. Three criteria were used to screen articles for relevance: Firstly, we only selected original research articles that presented empirical data. Secondly, we only selected articles describing research whose field methods could have been performed by citizen scientists. We defined this as excluding the use of any kind of specialized equipment for monitoring other than cameras, binoculars and butterfly nets. Essentially, this restricted the scope of the studies selected to the investigative scales of population and community ecology, although behavioural ecology studies were also included where they made use of passive field observations. Lastly, to keep the focus of analysis on UE, we excluded research for which observations or fieldwork were conducted *only* in non-urban or pristine environments. Thus, studies that compared rural and urban sites were included, as were studies of municipal nature reserves, remnant woodlands surrounded by urban matrices, and mixed residential landscapes. Following Macgregor-Fors [34], we included study sites described as ‘peri-urban’ where significant inter-mingling of urban and non-urban landscape types was noted, and excluded ‘ex-urban’ sites as places removed from the direct influence of urban cores. Golf courses were included where they were located in peri-urban or intra-urban areas.

The final dataset consisted of 562 papers for birds and 61 papers for butterflies.

### b. Tagging criteria

We developed a three-tiered hierarchical scheme for categorising UE research themes encountered according to the scope of this review. Studies were first assessed on their analytical scale; those that focused more on collecting species-specific data other than occurrence, and which did not aggregate the species surveyed for analysis, were tagged as representing the ‘Species’ category, whereas studies that collected occurrence and abundance data for more than one species per survey point, and/or that aggregated the species surveyed for analysis, were tagged as representing the ‘Community’ category. The second level corresponded to the research domain of the study (Domain), whereas the third level provided finer thematic resolution on the specific hypotheses/aims of the study (Category). Through an iterative process, 61 categories belonging to 19 research domains were identified (Table 2). The categorisation of domains and themes was performed collectively by all the authors after the full suite of papers meeting the inclusion criteria for this review had been identified, following which a single author (JWW) tagged domains and themes to each of the studies to ensure standardisation.

**Table 2:** Description of 19 research domains and 61 research categories (sub-domains) identified from 624 scientific papers selected for review as relevant for the urban ecology of birds and butterflies, published between 2005 and 2014.

Domain	Category	Abbrev.	Definition
Analysis	Adaptive guilds	Anaada	Analyses the data with respect to the urban adaptation schematic of urban avoiders, urban adapters and urban exploiters.
	Ecological indicators	Anaeco	Analyses the data to derive ecological indicators of landscape condition
	Foraging guilds	Anafor	Analyses the data with respect to diverse foraging guilds
	Functional traits	Anafun	Analyses the data with respect to specific phenological traits
	Homogenisation	Anahom	Analyses the data with respect to community homogenization
	Nestedness	Ananes	Analyses the data with respect to quantifying nestedness of species site occurrence records
	Reproductive guilds	Anarep	Analyses the data with respect to guilds associated with reproductive site preferences, i.e. nesting guilds for birds and host specialization guilds for butterflies
	Residency guilds	Anares	Analyses the data with respect to resident/migrant guilds
	Species-area	Anaspe	Analyses the data with respect to species-area relationships
	Voltine guilds	Anavol	Analyses the data with respect to the number of generations or broods produced per year
Autecology	Urbanisation	Auturb	Describes species distribution and abundance in relation to their putative drivers in the context of urban colonization
Before-After Control-Impact (BACI)	Full	Bacful	Performs a full before-after control-impact study
	Partial	Bacpar	Performs a before-after study
Behaviour	Diet	Behdie	Describes diet of study species through scat/pellet analysis or *ad hoc* observations
	Flight initiation distance	Behfli	Describes flight initiation distance (for birds)
	Foraging	Behfor	Describes foraging behaviours, including predatory behaviour
	Predation evasion	Behpre	Describes predation-evasion behaviours
	Competition	Behcom	Describes aggressive/competitive behaviours either within or between species
Breeding	Macro	Bremac	Assesses the influence of a rural-urban land-cover gradient on breeding success rates
	Meso	Bremes	Assesses the influence of landscape configuration on breeding success rates
	Micro	Bremic	Assesses the influence of local habitat variables on breeding success rates
	Nesting habit	Brenes	Describes nest structures or construction behaviours
	Others	Breoth	Documents breeding success rates or instances
	Parasitism	Brepar	Assesses the influence of parasitism on breeding success rates
	Predation	Brepre	Assesses the influence of predation on breeding success rates
Community trends	Long	Comlon	Estimates trends in community metrics for more than a decade
	Short	Comsho	Estimates trends in community metrics for less than a decade
Competition	Macro	Commac	Describes competition between species inferred from species distribution patterns
Environment	Macro	Envmac	Assesses the influence of a broad land-cover gradient including both urban and non-urban areas
	Meso	Envmes	Assesses the influence of landscape configuration e.g. patch area, age, perimeter, isolation within urban areas only
	Micro	Envmic	Assesses the influence of local habitat variables such as floral abundance and diversity, canopy density and management intensity
	Seasonality	Envsea	Assesses the influence of seasonality on species distribution and abundance patterns
Exotic species	Autecology	Exoaut	Describes species distribution and abundance in relation to their putative drivers for non-native species
	Effects	Exoeff	Describes the effects of non-native flora or fauna species on native biota
Habitat fragmentation	Effects	Habeff	Describes the effects of habitat fragmentation in urban areas
	Mitigation	Habmit	Describes initatives to mitigate habitat fragmentation in urban areas
Human impacts	BWC	Humbir	Documents bird-window collisions
	Ecological traps	Humeco	Documents evidence for the existence of ecological traps in urban environments
	Feeding	Humfee	Documents impacts of human feeding on species populations in urban areas
	Habitat destruction	Humhab	Documents impacts of habitat destruction in urban areas
	Physical disturbance	Humphy	Assesses the impacts of human traffic on species distribution, abundance or breeding
	Restoration	Humres	Documents impacts of site-specific habitat restoration in urban areas
	Socioeconomics	Humsoc	Assesses the influence of socioeconomic factors on species distribution and abundance patterns or vice-versa
	Urbanisation	Humurb	Assesses broad-scale impacts of urbanisation (including human population density) on species distribution and abundance patterns
Method	Abundance	Metabu	Describes the development of methods for improving species abundance estimates
	Occurrence	Metocc	Describes the development of methods for improving species occurrence estimates
	Other	Metoth	Describes other survey methods
Movement	Edge shyness	Movedg	Describes edge movement behaviour in relation to habitat connectivity
	Foraging	Movfor	Describes foraging movements
Multi-taxa	Others	Muloth	Describes other studies reporting the abundance and distribution of multiple taxonomic groups
	Surrogates	Mulsur	Assesses the mutual surrogacy of diverse taxonomic groups in relation to habitat requirements
Phenology	Climate	Phecli	Describes the influence of climatic variations on general phenological observations
	Migration	Phemig	Describes inter-annual variations in migration phenology
	Urbanisation	Pheurb	Describes the influence of urbanisation on general phenological observations
Plant-animal interactions	Dispersal	Pladis	Describes seed dispersal by fauna
	Pollination	Plapol	Describes pollination by fauna
Population	Size	Popsiz	Estimates population size of species
	Trends- long	Poplon	Estimates species-specific population trends for more than a decade
	Trends- short	Popsho	Estimates species-specific population trends for less than a decade
Species distribution modelling	Current	Specur	Parameterises species distribution models
	Simulation	Spesim	Simulates future species distribution models in the context of alternative scenarios of environmental change

We note that since the use of volunteers to collect ecological data pre-dates the use of the term ‘citizen science’, drawing conclusions about the thematic coverage of CS in bird and butterfly ecology based only on papers that included this term specifically could have severely underestimated the actual usage of such datasets [35]. Therefore, we determined a paper to have used CS if it reported the use of volunteer-derived data in any form (survey, questionnaire, and submitted observations) solely or in tandem with other empirical data, whether or not it was encountered through searching for either ‘citizen science’ or ‘urban ecology’.

To further identify associations between specific types of CS used in relation to the research themes identified above, we recognised two main types of CS contribution. Firstly, we categorised “primary” use in terms of whether the CS data was used for key analyses, and “secondary” use when it was relied upon as a supplementary but not indispensable resource. Secondly, we applied the framework for understanding the spectrum of options for public participation in scientific research identified by Shirk et al. [33] (Table 3). To differentiate between Contributional and Collaborative datasets, we determined a dataset to have been produced using a Collaborative mode if the program in question was developed and administrated by a non-academic institution; this included most breeding bird surveys (standardised sampling protocols conducted at the same locations to monitor species relative abundance over time). We considered atlas datasets (*ad hoc* records of species presences contributed by volunteers over variable spatial and temporal scales) to be Collegial in the sense that contributions were solicited on a case-specific basis.

**Table 3:** Five engagement modes describing possible relationships between scientists and citizen scientists in the development and implementation of citizen science studies, *sensu* Shirk et al. [33].

Contractual	Citizens ask scientists to conduct scientific investigation and report results
Contributional	Citizens are asked by scientists to collect and contribute data and/or samples
Collaborative	Citizens assist scientists in developing a study and collecting and analyzing data for shared research goals
Co-created	Citizens develop a study and work with input from scientists to address a question of interest or an issue of concern
Collegial	Citizens independently conduct research that advances knowledge in a scientific discipline

We calculated the contributions of CS to urban ecological research as the percentage of studies which used CS data overall for each taxon, as well as separately for each research category (Research aim 1). To characterise associations between the engagement mode and primary/secondary use paradigms of CS in relation to urban ecological research (Research aim 2), we used non-metric multi-dimensional scaling (NMDS). To determine whether the investigative scale of CS papers (species versus community-level) was different from that of the overall UE literature, we used Fisher’s exact tests.

To identify specific domains and categories with a higher potential for CS involvement (Research aim 3), we first used tree maps to identify the key categories whose proportional representation in the CS literature was most dissimilar to that found in the overall UE literature. Tree maps were useful for visualizing the overall allocations of categories between CS and UE for both domains and categories simultaneously; however, they did not identify categories with zero representation in CS. To account for this, and to quantitatively identify specific categories of UE for further investigation by CS, we calculated the standardised values (z-scores) of all categories by subtracting the mean and dividing by the standard deviation of the number of studies identified in the CS and UE datasets accordingly [36]. We then computed the difference between z-scores (UE–CS) to identify categories well explored by UE, but poorly explored by CS in comparison; the top ten categories identified by this approach were tabulated. The full lists of rankings are provided as supporting information (S3 Table and S4 Table) separately for birds and butterflies, while the complete lists of references and tags assigned are made available for reference (S5 Table and S6 Table). To provide more detailed resolution into the specific research questions which could be productively examined with CS approaches, the key findings of papers from these categories were reviewed, and topics on which less consensus was apparent from the UE literature over the time period examined were merged and highlighted for discussion.

All statistics and graphs were generated using R 3.1.2 [37], with the packages *treemap* [38] and *vegan* [39].

## 3. Results

The number of journal articles selected for urban bird ecology greatly exceeded those selected for urban butterfly ecology (562 vs. 61 respectively). For simplicity, the main groups and categories of research questions identified for both taxa are described together, and the main research themes of urban butterfly ecology are regarded as being (almost) completely nested in those of urban bird ecology [40].

### a. Citizen science contributions to urban ecology and their investigative paradigms

Overall, of the 623 journal articles on the urban ecology of birds and butterflies, 131 (21%) used citizen science datasets (Table 4). This proportion was marginally higher for butterflies (26%) compared to birds (20%).

**Table 4:** Parameters of CS contributions to UE for birds and butterflies combined (summed total, not weighted total) and compared for birds and butterflies. These are presented as the number of studies tagged in each category, followed by its proportional representation in the literature as a rounded percentage of either the total number of UE studies (rows 1, 7–8) or the total number of CS studies (rows 2–6). The tags applied for the rows marked with asterisks were not mutually exclusive; hence the percentages do not necessarily tally to 100.

No.	Dimension	Overall (%)	Birds (%)	Butterflies (%)
1	UE papers which used CS data	131 (21)	115 (20)	16 (26)
2*	Used CS data in Collegial mode	56 (43)	49 (43)	7 (44)
3*	Used CS data in Contributional mode	39 (30)	34 (30)	5 (31)
4*	Used CS data in Collaborative mode	42 (32)	38 (33)	4 (25)
5	Used CS as primary data	118 (90)	102 (89)	16 (100)
6	Used CS as secondary data	13 (10)	13 (11)	0 (0)
7*	UE studies community scale	390 (63)	340 (60)	50 (82)
8*	CS studies community scale	82 (63)	72 (63)	10 (63)

**Fig 2:**
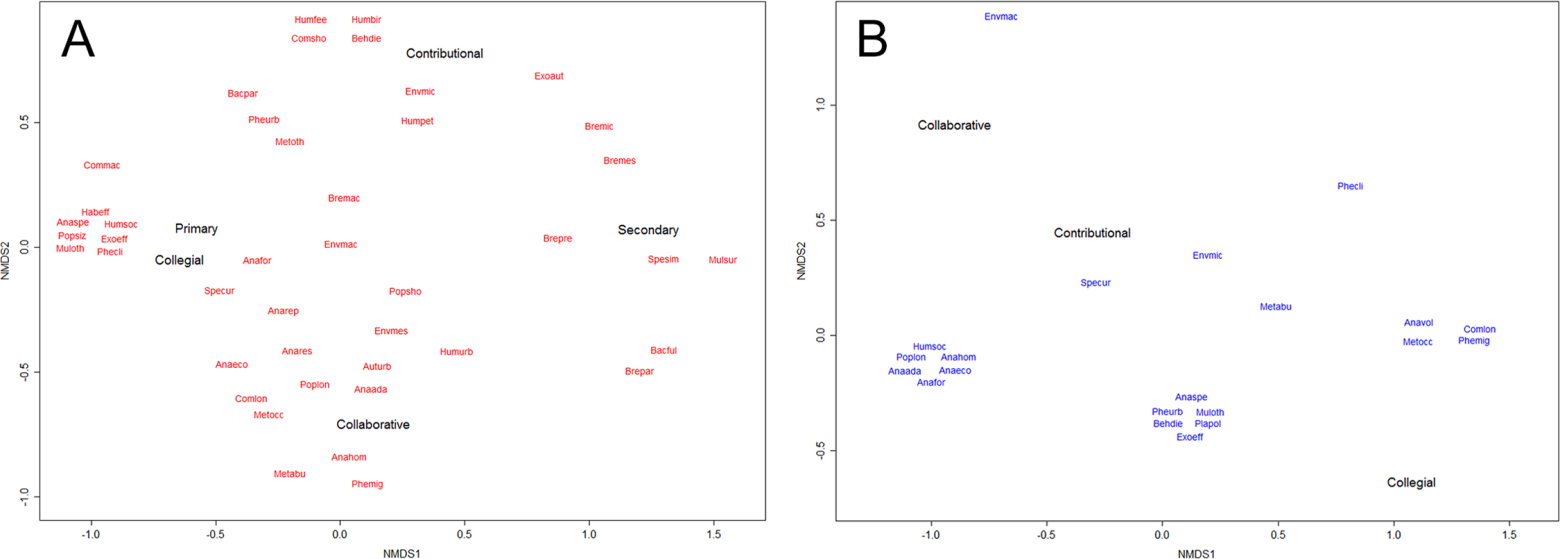
**Non-metric multi-dimensional scaling plot suggesting associations between thematic categories and citizen science usage and engagement modes for (a) birds and (b) butterflies**.

Most studies that employed CS used data collected in the Collegial engagement mode (43%), while reliance on the Contributional and Collaborative modes was approximately equal. This pattern was slightly different for butterfly studies, which showed roughly equivalent contributions from Collegial and Contributional modes. Two clarifications are needed for interpreting the relationships between engagement modes and thematic categories shown in Fig 2. Firstly, the tight clusters of research categories reflect categories represented by single studies, therefore the putative associations with citizen science modes for these categories should treated carefully. Secondly, for butterflies, the associations indicated by the second dimension do not faithfully match the data despite the NMDS registering a low stress metric (<0.02), possibly due to the low total number of citizen science studies for butterflies identified over the decade examined (n = 16). With these caveats, there was a tendency for engagement mode to be associated with thematic categories according to spatio-temporal scales of investigation across both taxa (Fig 2). The Collegial mode was associated with research themes with regional scale foci such as species distribution modelling and interspecific competition for birds, and phenological studies on migration and climate for butterflies. The Collaborative mode grouped mainly with long-term population analyses for both taxa, and method studies and guild analyses for birds. The Contributional mode was affiliated to research categories with more local (site-scale) foci such as BACI studies, impacts of human feeding, pets, bird-window collisions and micro-environmental influence for birds, and diet for butterflies. Two possible points of difference between the taxa were the lack of Collegial studies used for generating species distribution models for butterflies compared to the dominance of this mode for birds, and the greater emphasis on Method studies in Collaborative modes for birds compared to butterflies. We did not find any studies which used CS data collected in either Contractual or Co-created modes according to our interpretation of Shirk et al.’s framework [33].

The vast majority of papers which used CS data used it for primary analyses (90% overall), and this trend was more pronounced for butterflies (100%) compared to birds (89%). For birds, certain thematic categories displayed a greater tendency to use CS data in a supplementary manner. These included breeding studies as well as those investigating surrogacy of environmental responses between multiple taxa and species distribution modelling (Fig 2A).

Using Fisher’s exact tests, there was no evidence of a difference in focus in terms of ecological scale (community vs. species) between CS studies and the overall UE literature either overall (*p* = 0.92), or separately for birds (*p* = 0.84) or butterflies (*p* = 0.17), although proportionally more butterfly UE studies addressed community-scale question compared to CS studies.

### b. Representation of thematic categories between the UE and CS literature

**Fig 3:**
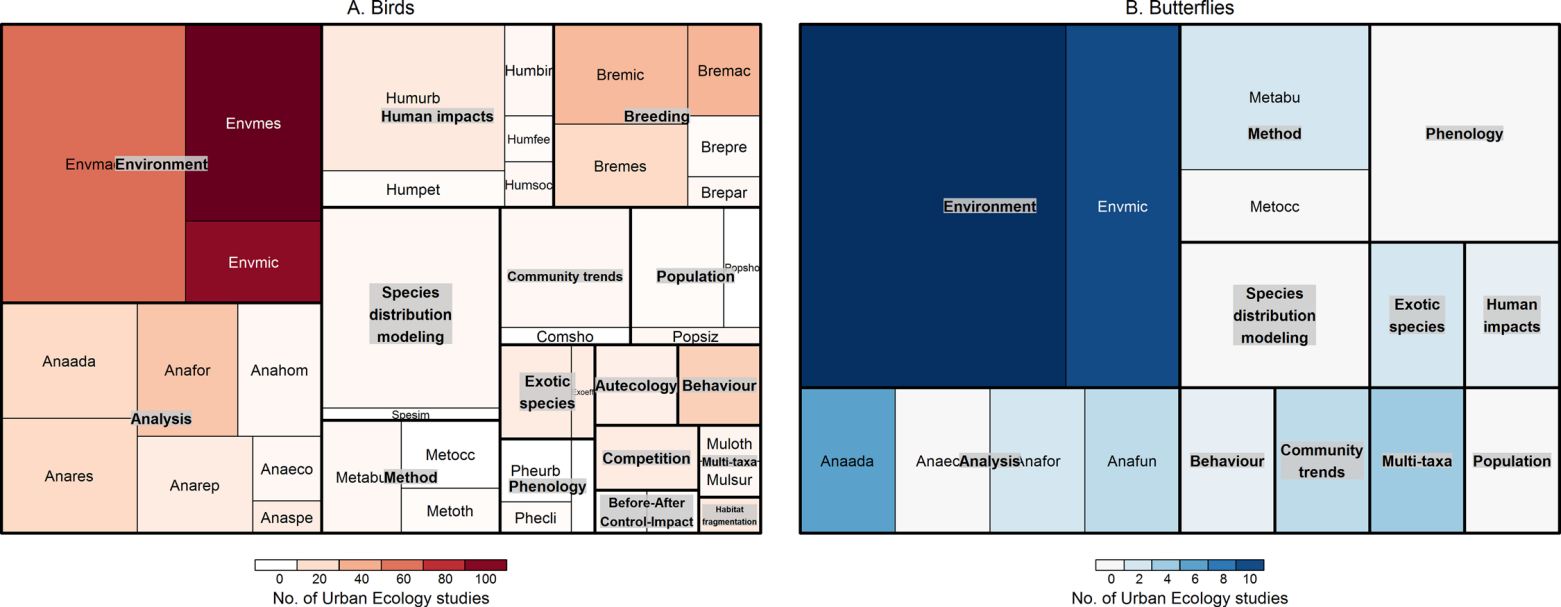
**Hierarchical tree map contrasting the relative popularity of research theme categories addressed with CS datasets to that of the wider UE literature for birds (a) and butterflies (b):** the size of the boxes represents the relative popularity of each category amongst CS datasets, while the shading represents the relative popularity of each category out of the overall UE dataset.

A general observation was that studies on species-environment relationships were under-represented in the CS literature compared to the UE literature for both taxa (Fig 3, Table 5). However, the influence of environment at meso scales was the more under-represented category for butterflies, whereas the microenvironment influence was the more under-represented category for birds. Environmental influence in relation to seasonality was equally under-represented for both birds and butterflies. A second group of categories which tended to be under-represented for both taxa were behavioural studies; specifically, those relating to diet, foraging, movement and response to human presence (Flight Initiation Distance). For birds, breeding studies in general were underrepresented in the CS literature (Fig 3A), whereas for butterflies only, multi-taxa studies and adaptive guilds were underrepresented (Fig 3B). To streamline the subsequent discussion, some categories in Table 5 were merged; namely, Movement: foraging was affiliated to the Behaviour domain, Habitat fragmentation: effects was affiliated to Environment: meso, and Human impacts: physical disturbance was affiliated to Environment: micro. The identification of topics for further CS investigation excludes topics shown in Table 5 which were assessed to be not feasible for CS involvement due to the continuous time commitments involved; namely, Environment: seasonality for both taxa, and Autecology: urbanisation for butterflies.

**Table 5:** Top ten research categories under-represented in the CS compared to UE literature for birds and butterflies, ranked by the difference between z-scores for UE and CS respectively.

	Birds		Butterflies	
Rank	Category	Z_UE_−Z_CS_	Category	Z_UE_−Z_CS_
1	Environment: micro	3.39	Environment: meso	4.21
2	Environment: meso	2.41	Habitat fragmentation: effects	1.23
3	Environment: seasonality	1.10	Environment: seasonality	0.73
4	Breeding: macro	1.04	Analysis: adaptive guilds	0.70
5	Behaviour: foraging	0.96	Environment: micro	0.63
6	Behaviour: diet	0.64	Autecology: urbanisation	0.48
7	Human impacts: physical disturbance	0.52	Behaviour: foraging	0.48
8	Behaviour: flight initiation distance	0.44	Human impacts: physical disturbance	0.48
9	Habitat fragmentation: effects	0.42	Movement: foraging	0.48
10	Behaviour: competition	0.39	Multi-taxa: surrogates	0.48

Research categories well-exploited by CS approaches were similar for both taxa; namely, environmental influence over direct urbanisation gradients, and species distribution modelling (Fig 3). Specifically, for butterflies, many phenology studies also employed CS data (Fig 3B).

## 4. Discussion

### a. Key findings

Citizen science data were used in approximately one-fifth of all journal publications on the UE of birds and butterflies that could have employed CS methods over the last decade. This is surprising, considering that CS biodiversity research is still considered a developing paradigm. Other studies that have documented the scientific outputs of CS programmes have done so from an administrative, rather than a methodological, perspective. For example, Theobald et al. [4] reported that 12% of 388 biodiversity-focused CS projects were associated with at least one peer-reviewed publication, whereas Tulloch et al. [5] found that breeding bird survey programmes were associated with a higher number of publications per program compared to atlas programmes. Although not all studies which could possibly involve CS will necessarily benefit from doing so, the sizeable fraction of publications which have use CS data suggest that at least for several key research domains, the potential benefits of CS are being exploited well. Nevertheless, given that most research domains and categories were not well-explored using CS data implies many opportunities for knowledge gain through more targeted applications of CS.

A second key finding of this review was that certain research themes that were heavily explored in the UE literature were very poorly explored using CS for both taxa; namely, questions relating to the environmental factors influencing species ecologies in urban landscapes. Several reasons are proposed for this general pattern, which could also apply for other taxa. Firstly, many CS datasets provide regional distributional data of only indirect relevance to drivers of species diversity at landscape to habitat scales. Secondly, most of these datasets generally only provide primary data on taxa species richness and abundance, without ancillary data for correlation. At landscape scales, the proliferation of archived satellite imagery enables such studies to be conducted retrospectively, and these opportunities should be more widely exploited. Collecting ancillary data at the micro scale, including data on physical disturbance by humans, requires more planning and a greater commitment from field workers. This is where citizen scientists can work alongside professional ecologists through a partnership in which citizen scientists are trained and entrusted to collect good quality primary data, while ecologists focus on collecting the secondary data requiring greater technical expertise.

Nevertheless, one should consider taxonomic differences, which determines how CS programmes are structured. For example, we found that CS contributions to understanding urban environmental influence on birds and butterflies were reversed between meso and micro spatial scales. This possibly reflects differences in methodological requirements for micro-environmental studies between the two taxa: whereas butterflies are generally recognised to be sensitive to floral abundance and diversity, including the presence of host plants, birds are known to respond additionally to various characteristics of habitat structure such as canopy cover, foliage height diversity and substrate, which are more technical and time-consuming to measure. CS involvement in breeding studies could also be more relevant for taxa with more conspicuous breeding habits. For butterflies, the only studies which directly quantified reproductive effort were comprehensive autecological monographs requiring continuous observations over the reproductive season, which would clearly be impractical for CS. These considerations suggest specific reasons for technical constraints on volunteer involvement varying between taxa; professional inputs could be structured accordingly.

The slightly greater proportional representation of CS in butterfly ecology studies compared to birds is interesting from the perspective that the actual number of CS projects focused on birds far exceeds that on butterflies (Lepidoptera) [4], and even more so considering that the butterfly CS literature was dominated by Collegial mode of engagement characterised by research initiated independently by non-professionals (Section 4di). Although this phenomenon could be interpreted that butterfly CS projects are more efficient than bird CS projects in publishing their data, it is more likely that this reflects there being many more professional ornithologists than lepidopterists [4]; hence, it could have been expected for citizen scientists to make a more noticeable impact more quickly in this taxonomic category.

### b. Investigative paradigms of citizen science

Whereas the general merits of citizen science for investigating broad spatial and temporal scales have been recognised [3], and CS engagement modes have been analysed in relation to social and institutional needs [33], the matching of CS investigative paradigms to specific spatio-temporal domains is useful in terms of providing an *a priori* principle for identifying which mode of CS engagement may be best suited to particular research objectives. Based on the publication record, topics primarily focused on regional and global spatial patterns have benefited well from Collegial engagement modes, whereas questions focused on long term population trends seem well-suited to Collaborative modes. Ecological questions that are more focused in time and space appear to be most practically pursued within Contributional engagement frameworks.

It is interesting that the research themes investigated by the Contributional and Collaborative CS engagement modes were well separated for birds, but not for butterflies. This could be because the design of broad-scale butterfly surveys appears to have developed along a more integrated fashion, as compared to the clearer methodological bifurcation between Atlases and Breeding Bird Surveys for birds.

We found no Contractual CS studies in this review. This could reflect either the lack of incentive of civil societies to publish data collected by commissioned scientists, or concerns over data sensitivity, and so is most likely an artifact of the approach we adopted for paper selection. However, that no studies could be described as Co-created according to Shirk et al.’s definition [33] is more surprising. We surmise that the Co-created mode requires mature working relationships between both scientists and volunteers in terms of the levels of mutual trust and equivalence of competence in complementary aspects of programme development and maintenance. Thus, it may be that Co-created projects should not be expected to develop independently, but rather as more focused offshoots from Contributional or Collaborative programme structures.

Whereas no studies used CS as secondary data for studying urban butterfly ecology, those that did for birds used CS data on bird occurrence and abundance to investigate species-specific breeding parameters. The CS approach has clear merits for locating breeding sites of more quiescent species, but this secondary use could be adapted more widely to inform sampling stratification schemes investigating a much greater variety of research questions (c.f. later discussion on the merits of the Neighborhood Nestwatch Program).

Lastly, that there was no evidence that CS tended towards investigating either community-level or species-level phenomena compared to the overall distribution of investigative effort in UE in general suggests that CS contributions could be relatively insensitive to the ecological scale of analysis. This is a positive finding, suggesting that volunteer interest could be effectively channelled across a range of biological scales.

### c. Implications for the design of citizen science programmes

Considering that the practice of CS depends critically on modes of social organisation and administration available to programme managers in specific social contexts, it may often be the case that the engagement mode required for a CS project pre-empts and constrains the formal identification and codification of specific research questions. This is supported by the tendency for particular research categories to cluster around the different CS engagement modes (Fig 2). Therefore, the approach adopted in this section is to first highlight specific merits of existing programmes which could inform the design of new programmes or improvements to existing programmes, according to the various modes of engagement. These programme models set out general frameworks for programmes to support the investigation of as wide a range of research questions as may be practical. Nevertheless, their relevance for local applications should be considered concurrently and iteratively together with the identification of specific scientific questions of interest.

The second section of this discussion elaborates on specific research objectives that could benefit from more CS involvement based on differences in z-scores of their distributions in the CS and UE literature respectively. These were identified based on incidences of contrasting findings from the same research questions encountered in the specific subset of UE screened by the authors over the course of this review. We note that these objectives are not intended to map out general research directions for urban ecological (and biological) research as a whole, for which we refer to the reader to recent reviews by McDonnell & Hahs [41] and Shwartz [42].

### Model programmes in relation to engagement modes of CS

Collegial CS: for birds, the research categories investigated by Collegial-mode CS were clearly grouped into four categories that mostly enjoy high CS involvement, namely Competition: macro, Analysis: foraging and Species Distribution Modelling: current. Practically all Collegial-mode CS studies identified for urban bird ecology were Atlas projects, of which several contributed at least two papers: (1) Birdlife Australia Atlas, (2) eBird, (3) Spanish Atlas of Breeding Birds and (4) New York State Breeding Bird Atlas (NYSBBA), (5) Ontario Breeding Bird Atlas (OBBA). Of these, the OBBA provides the most parameters (occurrence, breeding evidence and point-count abundance), whereas the NYSBBA does not report abundance measures but is conducted on a finer-resolution grid (5x5 km cells). Tulloch et al. [5]’s recommendation for Atlas projects to incorporate finer spatial and temporal resolution to maximise their scientific impact applies particularly for Atlases with urban foci, where the spatial extent is restricted but spatial turnover in land-cover composition is significantly more rapid. Other studies have reviewed methodological aspects of Atlas design in depth [43–45], so we limit our comments here to suggest that urban Atlases consider the OBBA and NYSBBA specifically for practical guidance on improving spatio-temporal resolution and parameter collection to support the investigation of a broader range of research categories.

For butterflies, two distinct sets of categories have been explored by citizen scientists using the Collegial mode: phenology studies (Phenology: migration & Phenology: climate), and Behaviour: diet. Among the butterfly monitoring projects represented by contributions to scientific journals, the survey model developed by the Chicago-Illinois Butterfly Monitoring Network is arguably the most commendable in terms of producing data which could contribute to a greater variety of research categories, in particular long-term population trends [46]. In comparison, if collection of phenology and species distribution data is of primary concern, this could be optimised with a less structured data contribution model, as exemplified by the UK Phenology Network [47] and the Massachusetts Butterfly Club [48]. Finally, the collection of data on (adult) butterfly diet using the Collegial mode has recently been pioneered by the French Garden Butterfly Observatory [49,50], and represents a promising model for collecting primary data for studies on butterfly diet and plant-animal interactions, as well as secondary data for assigning butterflies into foraging guilds to support other ecological analyses.

Contributional CS: of the research categories which have been explored using the Contributional mode of CS for birds, those also identified as having a high potential for an expanded role for CS in general included Breeding: micro and Breeding: meso (Fig 3, Table 6). The Neighborhood Nestwatch Program (NNP) featured most prominently among this type of study as having potential for wider adoption in other urban areas. This programme is distinctive in that the most basic contribution made by volunteers is permission for scientists to make on-site observations on their properties; actual participation in the monitoring process is secondary [51,52]. This programme structure is an ideal model not just for studying breeding processes, but as a database of private sites likely to be receptive to hosting wildlife research in general. For example, Dowling et al. [53] used the NNP site list to select sites for their study on the influence of anthropogenic noise on bird song. There are no practical limits on the types of studies which could be hosted by cultivating a list of volunteer landowners supportive of biodiversity-type studies.

**Table 6:** Summary of research questions of potential value towards improving CS contributions to UE.

Category	Research questions
Environment: meso, Habitat fragmentation: effects	Which species traits are associated with sensitivity to matrix fragmentation or habitat loss respectively?
	How does isolation influence the habitat potential of an urban green space?
	How do temporal disturbance regimes affect species persistence in urban landscapes?
Environment: micro, Human impacts: physical disturbance	What is the status of plant-animal mutualisms in urban areas, and what implications do these have for the conservation of native flora?
	How do management regimes influence biodiversity in managed green spaces?
	What impacts do noise and physical disturbance have on urban wildlife communities, and how could these be minimized with design or management guidelines?
Behaviour: diet, foraging	What keystone resources exist for urban fauna meta-communities?
Breeding: macro, meso, micro	What factors influence productivity of species over broad urban environmental gradients?
	How should the provision of additional nesting sites (or host plants) be structured to facilitate reproduction?
Analysis: adaptive guilds Autecology: urbanisation	How could landscape or management interventions facilitate wildlife adaptation to urbanisation?
Multi-taxa: surrogates	What is the relative importance of various taxa to plant pollination?
	Which plant species support the most diverse range of pollinators?

Another theme associated with Contributional CS studies for birds was Environment: micro; two CS programmes that have successfully investigated this in the urban context are the Tucson Bird Count [54] and the CityRoots program [55]. The former represents a more structured, long-term approach, whereas the latter is a good example of the *ad hoc* approach to volunteer recruitment tailored to specific, time-bound project objectives.

For butterflies, among studies which used data generated using the Contributional mode, two are assessed to have a higher potential for supporting multiple angles of research: the Spipoll protocol of France [56], and the Backyard Habitat Program run by the National Wildlife Federation (USA). The merits of the latter scheme are similar to those described for the NNP, but are more strictly circumscribed to database provision rather than direct volunteer work.

Collaborative CS: the possibility of Collaborative CS contributing to more information on the influence of the environment on bird diversity at landscape scales (Environment: meso) is considered first; two of the few programmes that have successfully investigated this theme using Collaborative CS are the Ontario Breeding Bird Count [57] and the Perth Biodiversity Project [58]. The former adopted a distributed approach to abundance sampling using randomly distributed point counts, whereas the latter employed a discrete approach based on active search for presence/absence in selected metropolitan reserves. It is worth noting that any investigation of landscape-scale drivers of urban bird diversity will need to secure access to reliable land cover data, whether as planning layers [57] or raster data processed directly from satellite imagery [58].

For butterflies, reasonably clear associations between Collaborative CS and the categories Environment: macro [49,59] and Population trends: long [60] were apparent. Two Collaborative programmes that could be feasibly adapted to urban contexts include the French Butterfly Monitoring Scheme (FBBS, 185) and the Butterfly Monitoring Scheme of the Netherlands (BMSN, 162), the main difference between the two being randomised site selection for the FBBS, compared to free choice for the BMSN. However, neither of these Butterfly Monitoring Schemes are particularly well-suited for investigating urban environmental influence due to the coarseness of the environmental data specified in these protocols [61]. Urban butterfly monitoring schemes could benefit from adopting a more micro-scale focus in terms of recording floral abundance or plant stature such as in [62,63], while relying on data-specific land cover information from official sources.

### Research questions which could benefit from more CS involvement

Environmental influence at landscape scales (Environment: meso): studies investigating the influence of meso-scale (landscape) environmental variables on bird and butterfly diversity within urban areas were the most numerous of all the research themes for both taxa, but less than 10% employed citizen science overall, and none at all did so for butterflies (S4 Table). The role of habitat connectivity in structuring urban wildlife communities was a topic of enduring interest borne out in the studies reviewed here [63–67]. A key question of applied relevance to urban conservation is the relative influence of habitat area and configuration on meta-population persistence, yet there has been poor agreement on this topic from observation-based empirical studies over the last decade for both birds [65,68–70] and butterflies [63,71–74]. The more complete portrait offered by also considering radio-telemetry and landscape genetic studies suggests that the landscape needs of species could depend significantly on their life history traits [75,76]. Citizen science surveys may therefore be useful for identifying the species and life history traits most likely to benefit from improvements in matrix connectivity, or from buffering and expanding existing natural fragments in urban areas.

Besides habitat connectivity, two other promising research angles that could be productively examined with CS are the roles of isolation and age on urban wildlife communities. While isolation (e.g., distance to nearest habitat patch) is expected to exert a negative influence on species richness based on the island biogeography theory and this has been corroborated in some urban areas for both birds [69,77,78] and butterflies [64,74], there are also reports of species richness in parks being positively related to isolation, in what is suggested to be a ‘funneling’ effect [66,79]. This phenomenon should be investigated further in view of the implication that providing habitats in areas otherwise considered marginal for biodiversity may offer important benefits for wildlife conservation in cities. Thirdly, habitat age is postulated to influence urban biodiversity through anthropogenically-mediated vegetation succession in what is termed the ‘legacy’ effect [8], but evidence for this is also equivocal [80,81]. While urban areas are fundamentally characterised by rapid habitat turnover, a crucial question from a biodiversity conservation perspective is what spatial mix of disturbance regimes could support long-term species persistence in urbanised and urbanising landscapes. These questions could be directly addressed with well-designed CS datasets, but do require upstream planning and collection of land-cover and land-use data.

Environmental influence at microhabitat scales (Environment: micro): three major groups of urban microhabitat variables that have been investigated for their effects on bird and butterfly diversity involve structural, floristic and anthropogenic aspects. Of these, the influence of vegetation composition and green space maintenance regimes corresponding to the latter two topics could be considered more broadly applicable to a range of other taxa, since they are theoretically less dependent on organismal body size. Specifically, the question of how to balance conservation needs across both urban flora and fauna is one that deserves more sustained research attention. Although the basic association between plant species richness and faunal species richness may be intuitive and quite general, specific quantitative relationships identifying (novel) mutualisms between urban plants and animals are needed to effectively optimise urban landscapes for ecosystem-scale conservation. The general nature of this question renders it open to investigation with multiple modes of CS, from Collegial *ad hoc* observations of plant-animal interactions, to standardised surveys of individual trees or plant species in a contributory format.

Managed green spaces constitute a sizeable proportion of available habitat in urban areas, yet relatively few published studies have investigated how the maintenance regimes in parks and precinct greens could be affecting bird and butterfly diversity. Shwartz et al. [82] found that bird species richness was lowest in the most intensively-managed landscapes in a Mediterranean park, but also that it peaked at intermediate levels of management; a finding consistent with the intermediate disturbance hypothesis. Nevertheless, maintenance regimes are complex phenomena comprising different aspects of landscape management such as pruning, mulching, grass-cutting, fertilizing and pesticide use, and it is also reasonable to expect that these could affect various wildlife taxa in non-consistent ways [71]. The utility of applying a CS approach to study this question derives from the potential of expanding study sites to collect biodiversity information more comprehensively from urban green spaces varying according to management type and intensity, that may be best facilitated through a Contributional framework (e.g. [55]).

Anthropogenic disturbance from noise or human traffic could affect urban wildlife by limiting access to resources that could otherwise be more heavily exploited. These effects may be more taxon-specific. For example, there is evidence that anthropogenic noise negatively impacts urban bird populations [53,83–85], while there is less support for direct effects of human traffic [86,87]. For birds, flight initiation distance data could be useful for informing the size and width of buffer zones in urban parks required to support periods of undisturbed foraging [88], or for comparing with other anthropogenic factors to better explain and manage the negative associations observed between bird diversity and human and vehicular traffic [89,90]. Behavioural observations collected by citizen scientists from urban sites stratified by these anthropogenic pressures under a Contributional framework could provide practical information to guide the planning and management of urban habitats to balance these impacts against other conservation objectives such as public accessibility to natural spaces.

Behavioural studies (Behaviour: diet, foraging, movement): Although behavioural studies could in theory benefit from Collegial-style contributions from citizen scientists, a very low proportion of studies focused on urban bird and butterfly behaviours employed CS datasets (n = 4/59). Quantitative records of wildlife diet and foraging would be useful for understanding species responses to fine-scale urbanisation gradients [91] and pin-pointing keystone food resources [92,93]. Citizen science observations could contribute to understanding how wildlife may be adapting to novel conditions/resources prevalent in urban environments in locally idiosyncratic ways, such as the use and reliance on non-native nectar or host plants [94], facilitation of nocturnal foraging by night lighting [95,96], trapping of insect prey in glass buildings [91] and reliance on landfills as foraging sites [97,98].

The feasibility of citizen scientists collecting data on wildlife movements, specifically in relation to urban infrastructures and human disturbance, is quite likely to be limited to conspicuous diurnal animals. Nevertheless, the adaptability of diverse wildlife to anthropogenic environments may depend crucially on their ability to negotiate local barriers to resource use such as tall buildings [99], glass windows [100] and roads [101]. Aggregating observations of movements along these biotope boundaries through CS data could therefore contribute to understanding how built structures may be effectively re-designed to facilitate wildlife persistence from a behavioural ecology perspective.

Guild analysis (adaptive guilds): the avoider-adapter-exploiter framework developed by Blair [102] has since become a major heuristic guide for research investigating urbanisation effects for a variety of taxa. These typologies are emergent properties of species populations in response to specific environmental and biotic contexts, rather than reified species attributes. Understanding the mechanisms driving these population outcomes is important if long term species viabilities are to be sustained in urbanising landscapes. Citizen science observations could contribute more to this approach on at least two levels: firstly, *ad hoc* observations of how wildlife use urban landscapes for foraging [50] and reproduction [103], and secondly, by mapping the evolution of habitat associations of different species in response to urbanisation [104] and/or associated indirect factors such as food subsidies [105], exotic prey [106], predators [107] or competitors [108]. To the extent that species responses to urbanisation may be at least partially labile [109], knowledge of these mechanisms could inform management strategies aimed at expanding the adaptive range of as wide a suite of species as possible.

Multi-taxa studies (surrogacy): the quest to identify management surrogates [110], i.e. species whose management requirements broadly correspond to desirable aspects of ecosystem function, is another topic where CS efforts could make more substantial contributions. Although evidence is ambivalent as to the extent to which bird and butterfly diversity are mutual surrogates, or can surrogate other taxa [71,111–114], a promising paradigm to investigate surrogacy among multiple taxa is that of pollination ecology. Plants themselves could be considered surrogates for their pollinators, and are natural starting points for conservation interventions in urban landscapes. It could be cost-effective to flesh out pollinator networks supported by common urban plant species for multiple taxa by extending studies on the foraging preferences of butterflies or nectarivorous birds to other insect taxa which also visit the same flowers. Reliable photographic records sourced under a Collegial framework [56] could contribute significantly to enhancing urban pollinator diversity in cities generally by informing more judicious plant selection [93]. This line of enquiry could equally contribute much to our understanding of how to manage urban pollinator communities to support in situ urban plant conservation [115].

An important caveat is offered with respect to the research questions discussed above: the historical approach adopted above could offer limited insights towards prospects for the use of CS in UE over the long term, especially in response to developments in mobile technology and database infrastructure [116]. This is because the method for category selection we used focuses on historical disjunctions between CS and UE research; research themes which have recently emerged or equally un-represented in both CS and UE work would not have been identified. However, to the extent that research priorities are first identified by professional scientists before involvement of non-professionals is up-scaled, we suggest that the topics identified through this review could be relevant to the development of CS for UE in the near future.

## 5. Conclusion

The above discussion has considered potential avenues for expanding CS contributions to urban ecological questions that could be generalised beyond the two taxa examined. Substantial synergies of effort and outcomes exist where CS meets UE. This review is a first step in describing the character of the science at the interstices of these two disciplines, and mapped out the broad themes relevant to engaging meaningfully with both these two scientific domains at this point in their development. Urban ecologists can benefit by making more intentional and systematic efforts to design volunteer-led primary data collection, while amateur scientists and citizen volunteers have much to gain from pooling resources with each other and with professional ecologists. We hope that the synthesis of perspectives provided in this review will facilitate deeper dialogue and engagement between these young and promising fields of scientific enquiry.

## Supporting Information

S1 Table PRISMA checklist for systematic review. (DOC)

S2 Table Summary diagram of the methodological and logical framework applied in this review. UE: Urban Ecology, CS: Citizen Science, NDMS: Non-metric multidimensional scaling.
(XLSX)

S3 Table Citizen science and urban ecology metrics for birds. (XLSX)

S4 Table Citizen science and urban ecology metrics for butterflies. (XLSX)

S5 Table Full list of references and tags for birds. (XLSX)

S6 Table Full list of references and tags for butterflies. (XLSX)
